# Supplementary material for: Loss of multiple micro-RNAs uncovers multi-level restructuring of gene regulation in rodents
Source: BMC Genomics. 2025 Sep 2;26:800. doi: 10.1186/s12864-025-11815-3 (PMC12403560; doi:10.1186/s12864-025-11815-3)
Supplement: Supplementary file 2 — Supplementary Material 2. [file 12864_2025_11815_MOESM2_ESM.docx]

# Supplementary Figures


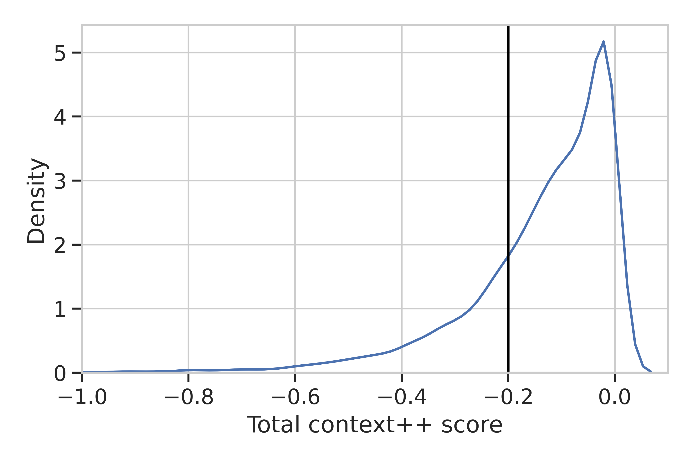


Supplementary Figure S1: Distribution of total context++ scores of miRNA targets predicted with TargetScan7. The minimum score threshold used for finding primary miRNA targets of -0.2 is marked with a black line.


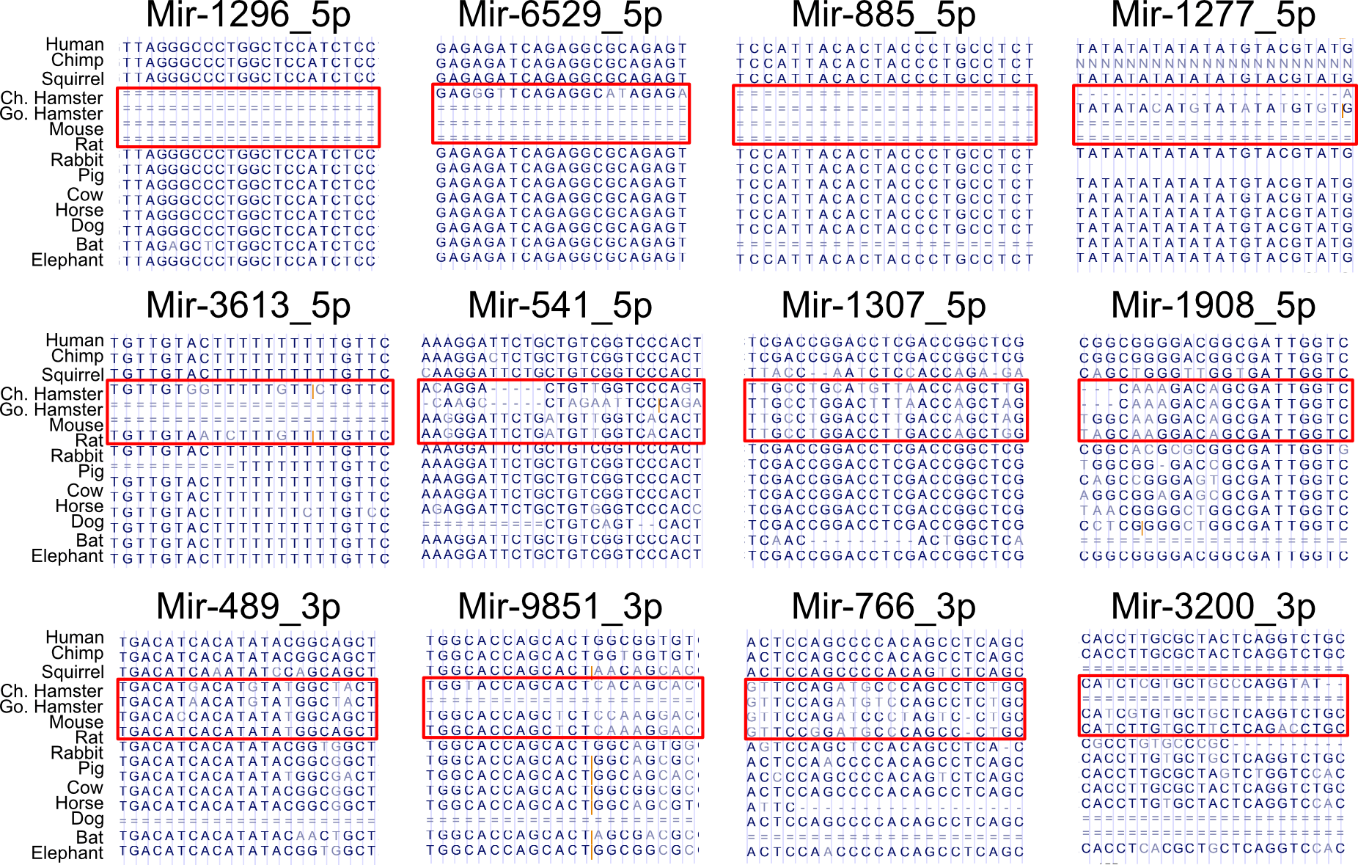


Supplementary Figure S2: 14-species whole-genome alignments of miRNA families lost the Eumuroidea. Eumuroidean species are marked with a red box.


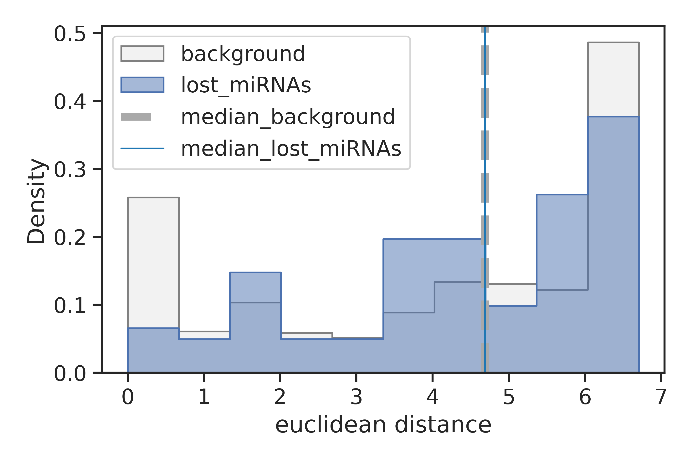


Supplementary Figure S3: Pairwise distances of miRNA expression vectors across 45 human tissues. Normalized reads of miRNA expression were downloaded from MirGeneDB 2.1 (34). A minimum threshold of 15 was set to determine presence or absence of a miRNA in a tissue. If a miRNA family was represented by multiple genes, the gene with the highest expression in the family was chosen as representative.


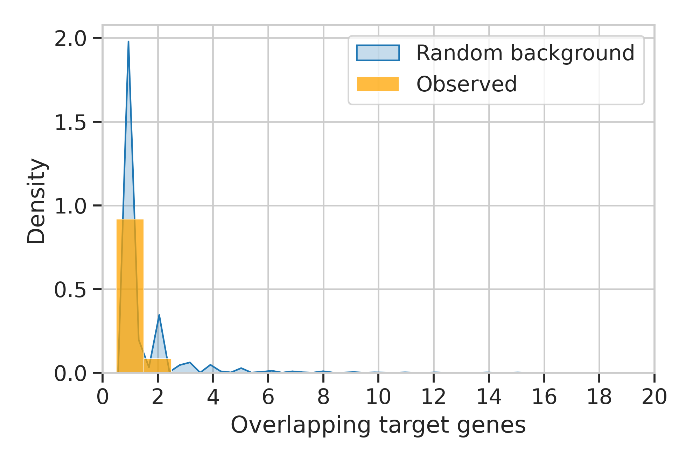


Supplementary Figure S4: Overlap of miRTarBase targets of lost miRNAs.


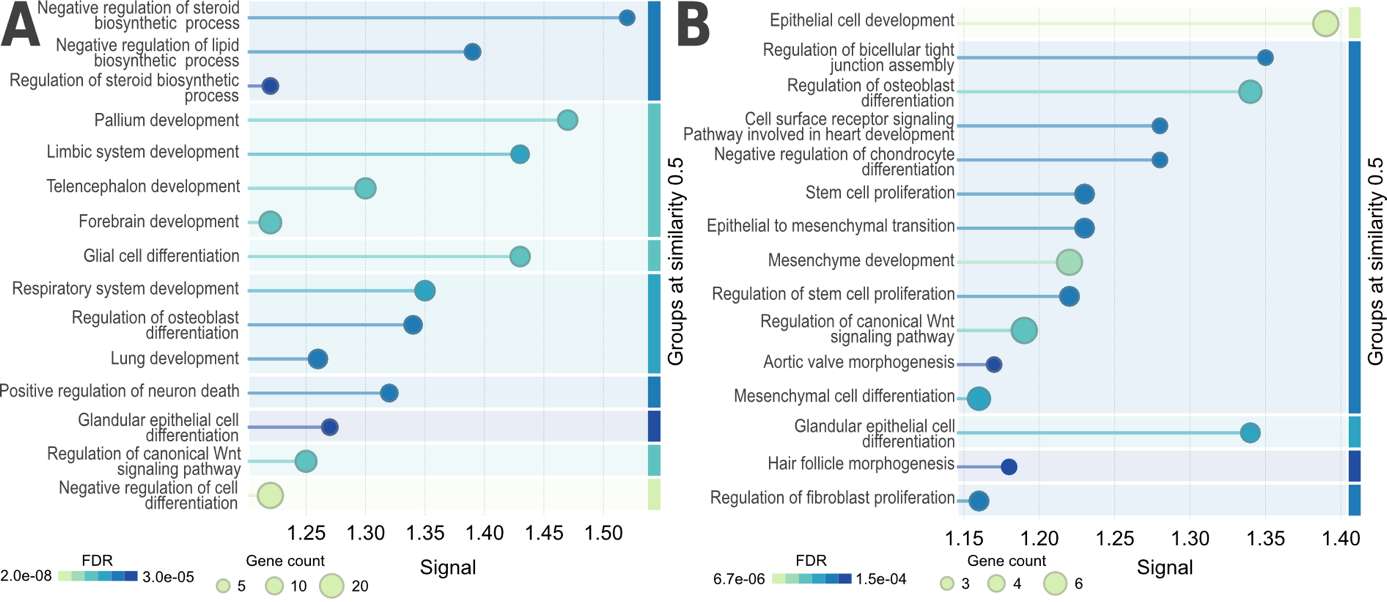


Supplementary Figure S5: Significantly enriched GO-terms of miRTarBase targets of lost miRNAs. Functional enrichment of biological process GO-terms was performed with STRING (38) using either all 73 reported targets of lost miRNAs in miRTarBase (A) or only the 24 targets of Mir-506 (B). STRING defines the “Signal” shown on the x-axis as the weighted harmonic mean between the observed/expected ratio and -log(FDR). The 15 terms with the largest signal are shown, for all other settings the default values were used.


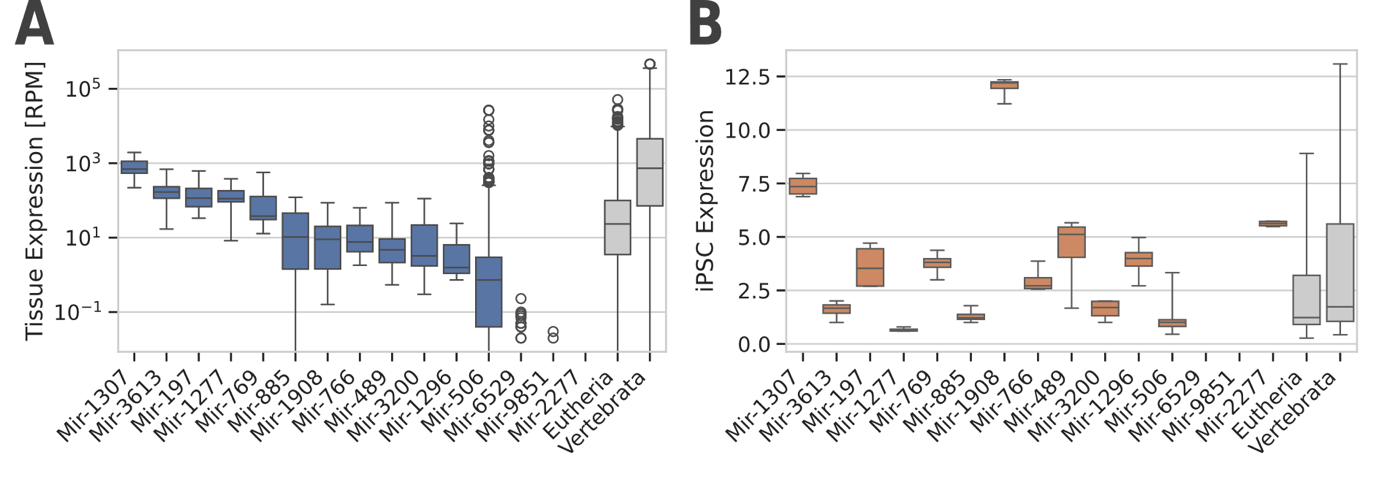


Supplementary Figure S6: Expression of miRNA genes lost in the Eumuroidea. Compared to all miRNAs gained in the LCA of Eutherians or at least as old as the LCA of the Vertebrata. A) RNAseq data of 45 tissues from MirGeneDB (34). B) Microarray expression levels in human iPSCs reported by Kulcenty et al. in 2019 (56).


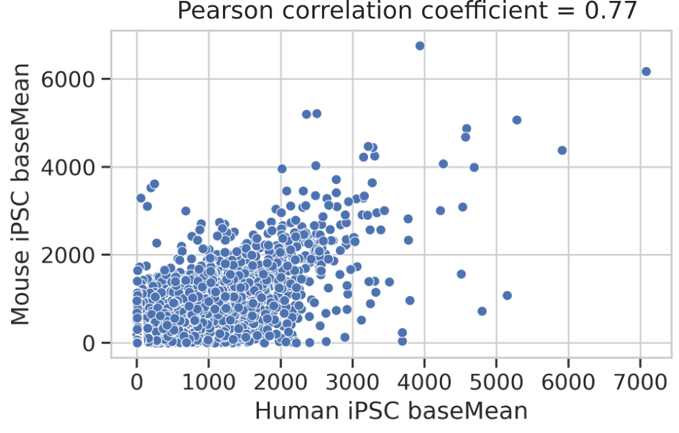


Supplementary Figure S7: Correlation of human and murine iPSC expression values. The p-value of pearson correlation coefficient is < 0.001.


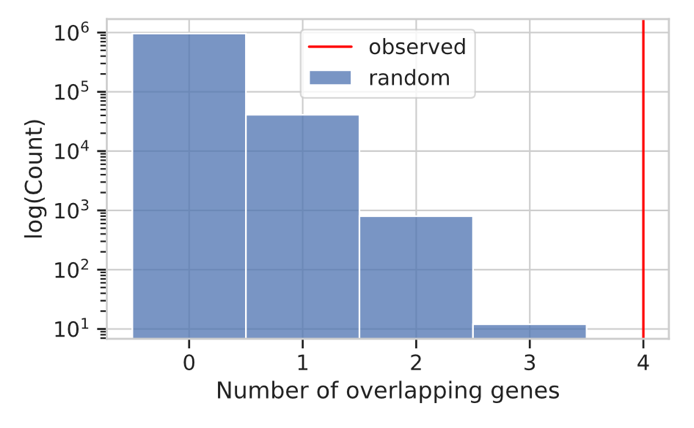


Supplementary Figure S8: Empirical assessment of the observed overlap between Mir-197 targets in human and murine iPSCs. Random sampling of 20 and 25 genes from the 9,402 and 8,456 genes expressed in human or murine iPSCs, respectively. The observed overlap of four genes (indicated by the red line) was not reached in any of the 1,000,000 iterations (empirical p-value < 1E-6). In a Fisher’s exact test the observed value amounts to a 75.2- or 67.7-fold enrichment (depending on the direction of the comparison) with p-values of 1.3E-9 and 2.2E-9, respectively.


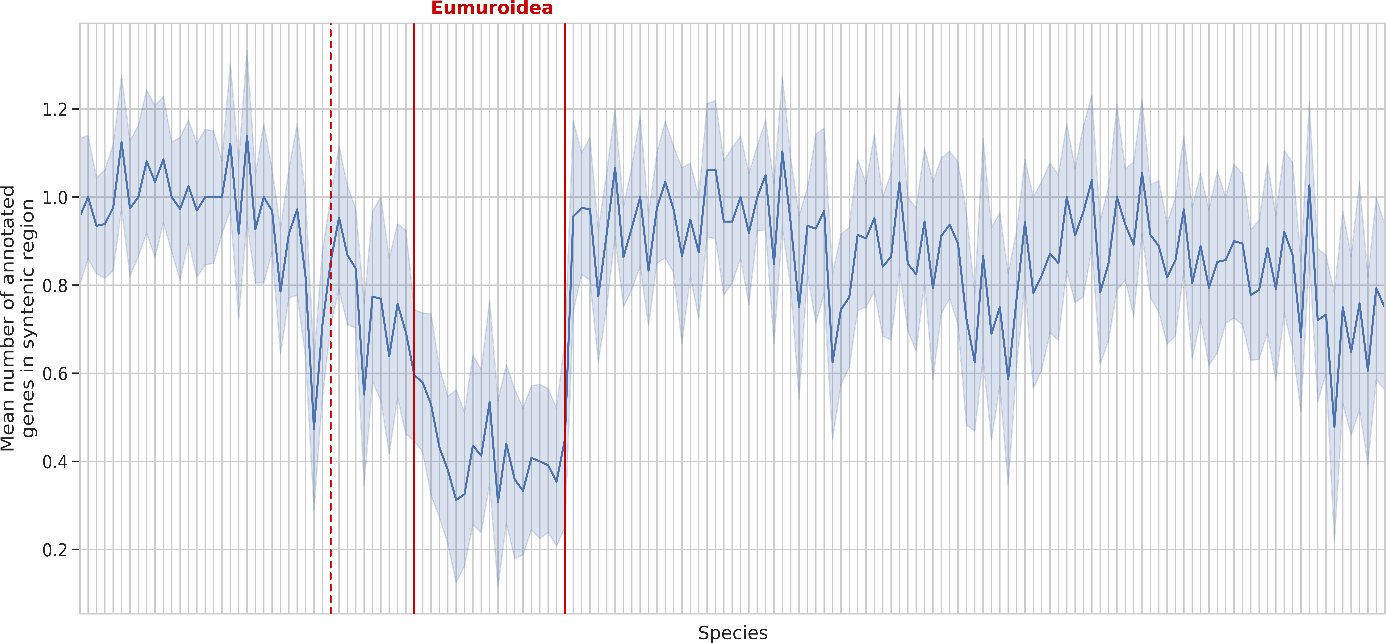


Supplementary Figure S9: Number of annotated genes in genomic region that is shared syntenic to human reference proteins. First, the number of annotated genes in all identified syntenic regions was counted. In taxa where no ortholog was found (i.e. the Eumuroidea), this value drops below 1, indicating that there is no annotated gene in the respective region.


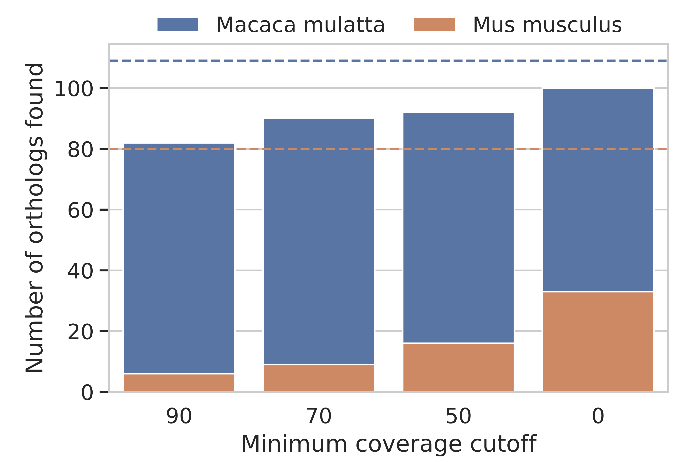


Supplementary Figure S10: Sequence similarity search identifies ruins of lost genes. A tBLASTn search of the human protein-sequence was performed in the respective shared syntenic region of the mouse genome. Number of identified syntenic region per species are marked with a dashed line. This identified short fragments that share a significant sequence similarity to the lost genes. Thus, the syntenic regions in mouse still harbour remnants of the lost genes but they are sufficiently degraded so that no gene was inferred in the region. This strongly indicates that the genes were indeed lost (57), and that the functions encoded by them are most likely absent in the Eumuroidea.


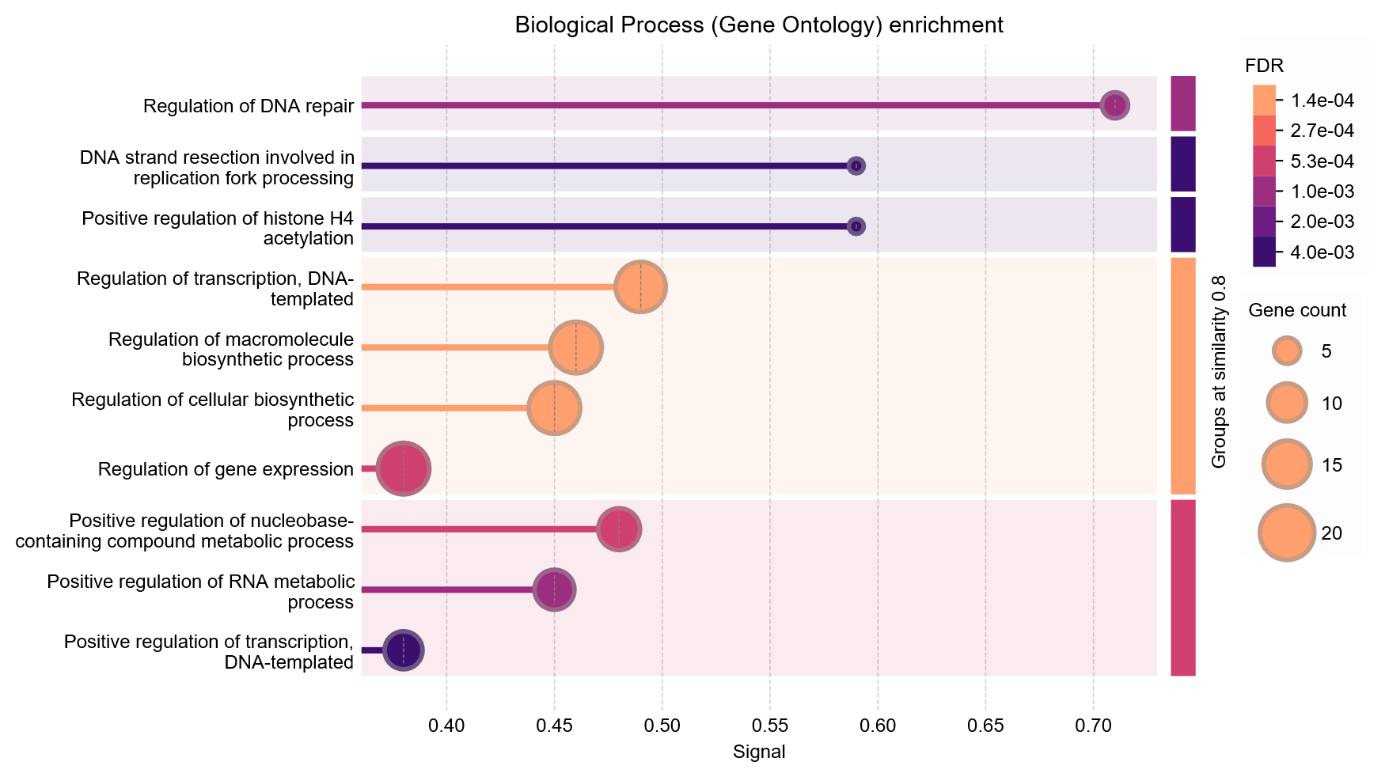


Supplementary Figure S11: GO-Enrichment analysis of the genes targeted by TFs lost in the Eumuroidea. Performed by the STRING database (38).


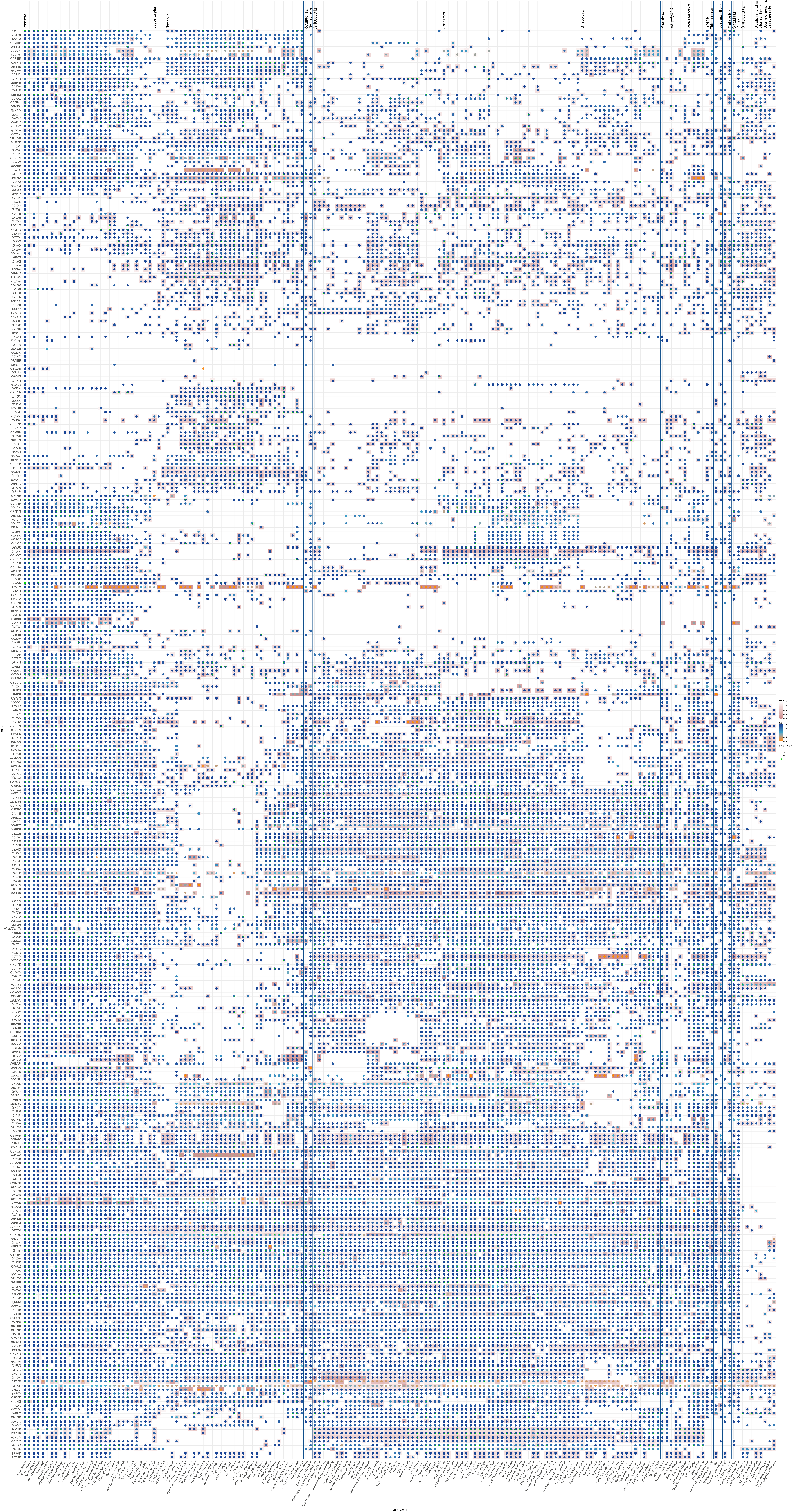


Supplementary Figure S12: Phylogenetic Profile of non-conserved human transcription factors.
